# Evaluating the Impact of COVID-19 on the Adoption of Virtual Care in General Practice in 20 Countries (inSIGHT): Protocol and Rationale Study

**DOI:** 10.2196/30099

**Published:** 2021-08-26

**Authors:** Ana Luísa Neves, Edmond Li, Alice Serafini, Geronimo Jimenez, Heidrun Lingner, Tuomas H Koskela, Robert D Hoffman, Claire Collins, Davorina Petek, Ana Claveria, Rosy Tsopra, Greg Irving, Gustavo Gusso, Braden Gregory O’Neill, Kyle Hoedebecke, Sandra Milena Espitia, Mehmet Ungan, Katarzyna Nessler, Vanja Lazic, Liliana Laranjo, Ensieh Memarian, Maria Jose Fernandez, Saira Ghafur, Gianluca Fontana, Azeem Majeed, Josip Car, Ara Darzi

**Affiliations:** 1 Institute of Global Health Innovation Department of Surgery & Cancer, Faculty of Medicine Imperial College London London United Kingdom; 2 Local Health Authority of Modena Modena Italy; 3 Center for Population Health Sciences Lee Kong Chian School of Medicine Nanyang Technological University Singapore Singapore; 4 Department of Public Health and Primary Care Leiden University Medical Center Leiden Netherlands; 5 Center for Public Health and Healthcare German Center for Lung Research (DZL) / BREATH Hannover Hannover Medical School Hannover Germany; 6 Department of General Practice Faculty of Medicine and Health Technology, Tampere University Hospital Tampere University Tampere Finland; 7 Department of Family Medicine Sackler Faculty of Medicine Tel Aviv University Tel Aviv Israel; 8 Irish College of General Practitioners Dublin Ireland; 9 Department of Family Medicine Faculty of Medicine University of Ljubljana Ljubljana Slovenia; 10 Primary Care Research Unit Vigo Health Area Vigo Spain; 11 Galicia South Health Research Institute Vigo Spain; 12 Information Sciences to Support Personalized Medicine Centre de Recherche des Cordeliers, Sorbonne Université, INSERM Université de Paris Paris France; 13 Department of Medical Informatics, Hôpital Européen Georges-Pompidou Assistance Publique - Hôpitaux de Paris Paris France; 14 Health Research Institute Edge Hill University Ormskirk United Kingdom; 15 Department of Internal Medicine Universidade de Sao Paulo Sao Paulo Brazil; 16 MAP Centre for Urban Health Solutions Li Ka Shing Knowledge Institute St. Michael’s Hospital Toronto, ON Canada; 17 Department of Utilization Management Oscar Health Dallas, TX United States; 18 Colombian Society of Family Medicine Bogotá Colombia; 19 Department of Family Medicine Ankara University School of Medicine Ankara Turkey; 20 Department of Family Medicine Jagiellonian University Medical College Krakow Poland; 21 Health Center Zagreb Zagreb Croatia; 22 Westmead Applied Research Centre Faculty of Medicine and Health University of Sydney Sydney Australia; 23 Australian Institute of Health Innovation Macquarie University Sydney Australia; 24 Department of Clinical Sciences in Malmö Lund University Malmö Sweden; 25 Internal Medicine and Epidemiology Research Group Skane University Hospital Malmö Sweden; 26 Leiro Health Center Leiro Spain; 27 Department of Primary Care & Public Health School of Public Health Imperial College London London United Kingdom

**Keywords:** primary care, telemedicine, virtual care, digital-first models, quality of care, patient safety

## Abstract

**Background:**

In recent decades, virtual care has emerged as a promising option to support primary care delivery. However, despite the potential, adoption rates remained low. With the outbreak of COVID-19, it has suddenly been pushed to the forefront of care delivery. As we progress into the second year of the COVID-19 pandemic, there is a need and opportunity to review the impact remote care had in primary care settings and reassess its potential future role.

**Objective:**

This study aims to explore the perspectives of general practitioners (GPs) and family doctors on the (1) use of virtual care during the COVID-19 pandemic, (2) perceived impact on quality and safety of care, and (3) essential factors for high-quality and sustainable use of virtual care in the future.

**Methods:**

This study used an online cross-sectional questionnaire completed by GPs distributed across 20 countries. The survey was hosted in Qualtrics and distributed using email, social media, and the researchers’ personal contact networks. GPs were eligible for the survey if they were working mainly in primary care during the period of the COVID-19 pandemic. Descriptive statistical analysis will be performed for quantitative variables, and relationships between the use of virtual care and perceptions on impact on quality and safety of care and participants’ characteristics may be explored. Qualitative data (free-text responses) will be analyzed using framework analysis.

**Results:**

Data collection took place from June 2020 to September 2020. As of this manuscript’s submission, a total of 1605 GP respondents participated in the questionnaire. Further data analysis is currently ongoing.

**Conclusions:**

The study will provide a comprehensive overview of the availability of virtual care technologies, perceived impact on quality and safety of care, and essential factors for high-quality future use. In addition, a description of the underlying factors that influence this adoption and perceptions, in both individual GP and family doctor characteristics and the context in which they work, will be provided. While the COVID-19 pandemic may prove the first great stress test of the capabilities, capacity, and robustness of digital systems currently in use, remote care will likely remain an increasingly common approach in the future. There is an imperative to identify the main lessons from this unexpected transformation and use them to inform policy decisions and health service design.

**International Registered Report Identifier (IRRID):**

DERR1-10.2196/30099

## Introduction

### Background

Even before the COVID-19 pandemic, virtual care (a broad term that encompasses all the ways health care providers remotely interact with their patients) was on the rise, with many health care systems developing strategies to facilitate the adoption of this approach [1]. Yet, despite digital remote care having long been anticipated to play an increasingly important role in supporting primary care, its mainstream usage remained suboptimal and piecemeal in many countries, often limited by cultural, regulatory, industrial and technical, knowledge, financial, and market-related barriers, among others [2,3].

COVID-19 has brought an abrupt end to this unhurried introduction. Over the course of a short few weeks, primary care worldwide rapidly transitioned from face-to-face consultations to virtual care solutions [4,5]. In less than 1 year, virtual care approaches have taken center stage, triaging and monitoring patients with COVID-19 and other acute conditions in primary care, as well as ensuring access and continuity of care for those with long-term conditions (eg, diabetes, hypertension, asthma, psychiatric illnesses, chronic pain) [6-8]. Consequently, existing digital technologies and systems supporting virtual care suddenly faced an immense challenge, both in their ability to cope with the surge in use and the new myriad of clinical tasks they were now expected to fulfil. However, COVID-19 also presented a unique opportunity and a catalyst for furthering the deeper integration of virtual care into the modern primary care landscape [9,10].

One year on from this initial mass transition, there is a growing need to review the impact of widespread digital-first model usage on patients, carers, primary health care providers, and health systems worldwide. Much uncertainty remains surrounding whether systems now in place adequately address the diverse range of clinical needs found in primary care, as well as their effects on quality and safety of care delivery. Some efforts to promote remote care were based on the assumption that a considerable proportion of visits can be managed sufficiently remotely without compromising safety or quality of care [11]. However, this remains to be clearly demonstrated [12,13], and concerns have been raised regarding remote physical assessments [14]. It is also unclear whether the use of digital remote care will dissipate or attenuate after the pandemic is resolved [9] and whether general practitioners (GPs) and family doctors have the necessary training and support to comfortably deliver remote care [12,13]. Past research exploring this topic was largely built upon theoretical models such as the Technology Acceptance Model and Expectation Confirmation Model to gauge users’ acceptance or desire to continue using novel information technologies in relation to their perceived convenience and usefulness [15-17]. COVID-19 has provided an opportune moment to apply these conceptual constructs and assess whether they continue to hold true in real-life circumstances at scale and across varying primary care contexts worldwide.

As primary users of virtual care technologies, ascertaining feedback from GPs is imperative to understanding the extent of the use of these remote care technologies as well as evaluate their practical implications on quality and safety of care in order to inform guidance and policies concerning their continued use into the future. GPs’ experience during the pandemic is valuable as digital-first models no longer serve just as a backup to traditional face-to-face consultations, but rather act as an essential means for GPs to interact with their patients [18], among other reasons to protect the vulnerable and most sick, a role and responsibility not originally envisaged when virtual systems and technologies were first adopted. The lessons learned will likely outlast the pandemic and serve as a watershed moment in transforming how primary health care can be remotely delivered for decades to come.

### Aims

This study aims to explore GPs’ perspectives on the (1) use of virtual care during the COVID-19 outbreak, (2) impact of virtual care on quality and safety of care, and (3) critical factors for high-quality and sustainable use of virtual care technologies in the future.

## Methods

The study will use an online cross-sectional questionnaire completed by GPs. Online surveys have been successfully used in health care professional research and was chosen in this case to ensure widespread geographical coverage [19]. Recruitment started in June 2020 and was completed at the end of September 2020. The study adheres to the STrengthening the Reporting of OBservational studies in Epidemiology (STROBE) guideline for cross-sectional studies [20].

### Study Population

The inSIGHT Research Group is a collaborative research group of primary care researchers, aiming to explore the impact of the COVID-19 pandemic on the adoption of digital remote technologies in general practice/primary care. The research is conducted by a consortium spread across 20 countries (Australia, Brazil, Canada, Chile, Colombia, Croatia, Finland, France, Germany, Ireland, Israel, Italy, Poland, Portugal, Slovenia, Spain, Sweden, Turkey, the United Kingdom, and the United States).

### Sampling

Each local lead was asked to email an invitation to take part in the survey to GPs in their country, using their personal contact networks. The invitation included the participant information sheet as well as a link to the website hosting the survey. GPs were eligible for the survey if they were working mainly in primary care during the period of the COVID-19 pandemic. Recruitment of health care professionals during the COVID-19 pandemic posed significant challenges; additionally, low survey response rates are common in primary care [21]. Thus, local leads who had difficulty achieving the total minimum number (n=386) were asked to use snowballing, a recognized technique for recruiting hard-to-reach populations in health studies, to increase the number of responses [22,23]. Upon consenting to participate in the study, the survey remained active for the participant to complete for 2 weeks.

### Sample Size

The total population of GPs in the countries included was calculated using a combination of publicly available resources (Table 1).

**Table 1:** Proportionate allocation sampling estimate

Country	Total number of general practitioners (N=527,970)	Proportionate allocation sampling estimate (N=901)
Australia	36,938 [24]	63
Brazil	7149^a^ [25]	12
Canada	43,500 [26]	72
Chile	20,361 [27]	35
Colombia	522 [28]	2
Croatia	984 [29]	2
Finland	2362 [30]	5
Germany	47,708 [31]	82
Ireland	3378 [32]	5
Israel	5052 [33]	9
Italy	42,987 [34]	72
Poland	8439 [35]	14
Portugal	7768 [36]	14
Slovenia	1237 [37]	2
Spain	29,743 [38]	51
Sweden	6195 [39]	12
Turkey	24,082 [40]	42
United Kingdom	35,146 [41]	58
United States	204,419 [42]	348

^a^All doctors working in primary care settings were considered in this analysis, including those that had not completed formal training in general practice/family medicine.

Published response rates with medical practitioners are often lower than 30% [43]. Based on this estimated population and an expected response rate of 30%, sample size was estimated using the following formula, where N = population size, e = margin of error (percentage in decimal form), and z = z-score [44].



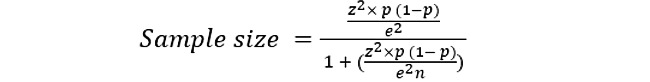



Anticipating an expected response rate of 30%, a total sample size of ≥901 responses was calculated to be sufficient to provide us with a confidence level of 95% and a margin of error of 5%. Sample sizes per country were determined via proportionate sampling calculations (Table 1). Under most conditions, fixed effects and their standard errors are unbiased; however, with fewer than 5 cases per group and fewer than 50 groups, standard errors for fixed effects will be too small (ie, increased Type I errors), and random effects (variances) and their standard errors may be underestimated. In a recent review, McNeish and Stapleton [45] suggested a minimum cluster size of 50, especially if fixed model estimation is used. Thus, to mitigate any potential issues of underrepresentation for smaller countries due to their resultant lower sample size estimates, all country leads were instructed to recruit a minimum of 50 participants to ensure a representative sampling of their GP population was collected. Given the 30 items in the questionnaire, this is also in excess of the rule by Hair et al [46] requiring a sample size to be at least 10 times that of the number of variables.



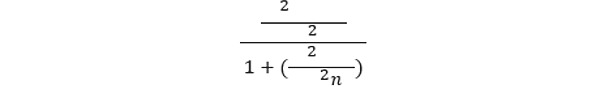



### Survey Development

The study was designed by investigators at the Patient Safety Translational Research Centre and Department of Primary Care and Public Health at Imperial College London (ALN, EL, GF, JC, AM, AD) in March 2020. The questions were generated from expert consultation, including both GPs and academics and researchers with experience in digital health and health services research. A draft survey was developed by the first author and subsequently reviewed by other co-authors prior to being finalized. The questionnaire (in English) was piloted by the national leads of the 20 inSIGHT Research Group member countries in May 2020 to identify statements that needed adaptation for cultural or organizational contexts at a national level. In order to improve participation rates, the questionnaire was translated into 5 additional languages (Portuguese, Spanish, French, Italian, and German) by inSIGHT Research Group local leads. Translation was carried out in a standardized way, with medically qualified native speakers of the local languages and fluent in English performing “forward” translations. The questionnaires were made available online using Qualtrics.

### Description of the Questionnaire

The questionnaire included an introductory section with participant information and 30 questions divided into 4 sections: (1) basic participant demographics, (2) use of digital-first models before and during the COVID-19 pandemic, (3) experience and impact of using digital-first models during the COVID-19 outbreak, (4) future of digital-first models in primary care.

The introductory section included the purpose of the study, a link to the participant information sheet, and an introduction to the concept of remote digital care (Multimedia Appendix 1). The first part included questions about participants’ individual characteristics (eg, age, gender, country, years of experience), as well as practice characteristics (eg, country; urban, rural, or mixed setting). The digital maturity of the practice was also evaluated using the Patient-Centred Framework for Evaluating Digital Maturity of Health Services [47].

The second part explored participants’ use and experience using virtual care approaches before and during the pandemic. Questions focused on the use of 8 virtual care solutions, selected after performing a rapid review evaluating the main tools used during the first months of the pandemic: (1) telephone consultations, (2) video consultations, (3) chat consultations, (4) online triage, (5) remote clinical monitoring technologies, (6) patient-initiated digital services (eg, scheduling, health education activities, prescription renewal, test requests), (7) secure messaging systems, and (8) remote access to electronic health records. Participants were asked about the availability and number of hours spent using each solution before and during the COVID-19 pandemic as well as the training, and guidance available or undertaken. In the third part, participants were asked about their overall experience and perceived impact of digital remote technologies on quality and safety of care. To evaluate impact on quality and safety of care, we adopted the definitions of the 6 dimensions of quality (ie, patient-centeredness, effectiveness, efficiency, safety, timeliness, and equity), as defined by the Institute of Medicine [48]. In this section, participants were also asked about the main benefits of, challenges to, and barriers to future use of digital remote technologies in primary care (free-text questions). The fourth and last section explored GPs’ perceptions on the future of digital remote models in primary care. In particular, participants were asked how they would like the adoption of these tools to evolve in the future and to identify the key aspects to ensure high-quality adoption of digital remote care in the future once the COVID-19 pandemic has resolved. A complete copy of the survey is provided in Multimedia Appendix 2.

### Statistical Analysis

For quantitative data, descriptive statistics will be calculated, including absolute (n) and relative frequencies (%) for categorical variables and mean (μ) and SD for continuous variables. At a first stage, differences in adoption of the various remote care technologies before and after the pandemic will be reported as the proportion of users before and during the pandemic. Potential associations with patients’ characteristics will be explored using Kruskal-Wallis tests and effect sizes calculated using Cramer v. If possible, we will explore the potential of using structural equation modelling (ie, partial least squares path modelling) to impute the relationships between unobserved constructs (latent variables) and observable variables [49]. The significance level for all statistical tests will be set at *P*<.05. 

For qualitative analysis, free-text answers will be analyzed; online surveys are a recognized qualitative research tool [50]. The framework analysis method was used, which includes 5 main stages: familiarization, identifying a thematic framework, indexing, charting, and mapping and interpretation [51]. The charting stage is applied as a principle for developing the coding framework through a process of abstraction to ensure that coding elements that might have been missed with an a priori approach are adequately captured [51]. Coding will be performed by at least two independent researchers, and the coding framework was kept both deductive and inductive, allowing the ongoing inclusion of emergent themes. All themes identified will be supported by quotations, and results will be presented both as conceptual maps and textboxes. Qualitative analysis will follow the Consolidated Criteria for Reporting Qualitative studies (COREQ) to ensure the study meets the recommended standards of qualitative data reporting [52].

### Dissemination

Sharing information about the project will take place throughout the duration of the work. Results will be published in peer-reviewed scientific journals as well as shared as preprints and in conference presentations. Local leads will be encouraged to publicize the project findings within their universities and health services, for example in newsletters, websites, meetings, and local journal publications. Additionally, patient partners will be included in the interpretation of our results, the co-development of a dissemination strategy, and summarizing the research findings into lay summaries and reports, in order to raise awareness and stimulate public participation on this topic.

## Results

Ethical approval was provided by the Imperial College Ethics Research Committee (ICREC) in April 2020. Data collection took place from June 2020 to September 2020. As of the submission of this manuscript, a total of 1605 GP respondents participated in the questionnaire. Both qualitative and quantitative data analyses are currently ongoing, with an anticipated publication date of the first results in mid-late 2021.

## Discussion

This study will provide novel insights into GPs’ perspectives on the availability of digital-first models before and during the COVID-19 outbreak. We will investigate their perceptions on their impact on quality and safety of care, as well as the critical factors surrounding high-quality, sustainable use of digital technologies in the future.

Despite the size and diversity of the sample, it remains a nonprobability, convenience sample that might have implications for representativeness, external validity, and overall generalizability of the study’s findings. As local leads recruited GPs at a national level using a range of methods, including convenience and snowballing sampling, there is an inherent risk of selection bias. Additionally, while online surveys have been successfully used in health care professional research and allow for widespread geographical and demographic coverage [53], their use also comes with possible selection bias, potentially favoring the participation of subjects who likely are more research-inclined and who have greater access to and are more familiar with digital technologies. It is also important to note that there is an unequal distribution of the number of GPs in total among countries (ie, high variance), which may introduce bias. The survey was translated in 5 languages, not in all the languages of the countries included in the survey, and the fact that it is not made available in the participant’s native language may influence both uptake and response behavior. Finally, it is important to consider that the questions evaluating the availability of digital-first solutions only capture self-reported availability rather than actual availability and thus are likely to introduce some level of reporting bias.

To our knowledge, this is the first study, both before and during the COVID-19 pandemic, to explore the availability of virtual care solutions in primary care and the perspectives of GPs regarding their impact on quality and safety of care at an international level. Being at the frontline of primary care delivery, GPs are ideally placed to identify the main pragmatic benefits and challenges of using digital tools for remote care as well as their impact on care quality and safety. Additionally, the study has included a large sample size, estimated based on sample power calculations and representing a variety of participants, health care settings and systems, and countries. An extensive description of the sample will be performed to explore the factors associated with the availability of remote care solutions and their perceived impact on quality and safety of care. Finally, the questionnaire was carefully developed and piloted with several national leads, capitalizing on their experience as frontline GPs working during the COVID-19 pandemic to ensure its relevance and ability to capture data necessary to address the study objectives.

The inSIGHT study will provide a comprehensive overview of the use of remote care technologies both before and after the onset of COVID-19 across 20 countries from the GPs’ perspectives, as well as attempt to capture the underlying factors in individual GP characteristics and the contextual characteristics in which they work, to better explain any findings observed. It will afford new knowledge about what digital tools worked well in the past and what is in dire need of improvement. While the COVID-19 crisis may prove the first great stress test as to the capabilities, capacity, and robustness of digital systems currently in use, remote care as a whole will likely remain an increasingly prevalent consultation method for years to come. It is therefore critical to reflect upon the main lessons to be learned from this global real-life experiment and capitalize on this transformative moment to improve the means upon which primary care will increasingly depend as we progress towards an increasingly digital future.

## Appendix

Multimedia Appendix 1 Participant information sheet.

Multimedia Appendix 2 Survey questions.

## Abbreviations

COREQ: Consolidated Criteria for Reporting Qualitative Research
GP: general practitioner
STROBE: Strengthening the Reporting of Observational Studies in Epidemiology
