# Supplementary material for: Evaluating the Impact of COVID-19 on the Adoption of Virtual Care in General Practice in 20 Countries (inSIGHT): Protocol and Rationale Study
Source: JMIR Res Protoc. 2021 Aug 26;10(8):e30099. doi: 10.2196/30099 (PMC8396553; doi:10.2196/30099)
Supplement: Multimedia Appendix 1 [file resprot_v10i8e30099_app1.pdf]

Imperial College London

Participant Information Sheet

Full Title of Project:

An International Study on the Impact of COVID-19 on the Adoption of Digital-First Models in Primary Care

Study Management Group:

Principle Investigator: Dr Ana Luisa Neves

Co-investigators: Dr Saira Ghafur

Research Invitation

You are being invited to take part in an online survey to help us assess the international impact of COVID-19 on digital-first models in primary care.

Before you decide it is important for you to understand why the research is being done and what it will involve. Please take time to read the following information carefully and discuss it with others if you wish.

Ask us if there is anything that is not clear or if you would like more information. Take time to decide whether or not you wish to take part.

Thank you for reading this.

What is the purpose of the study?

The aim of this project is to explore the international impact of COVID-19 on digital-first models in primary care through an online survey which will be completed by GPs.

The aim is to gain responses from a minimum of 8 countries (including but not limited to Portugal, Spain, France, Italy, Germany, Slovenia, Croatia, Israel, UK and Ireland) with at least 20 respondents per country, therefore the total number of participants in this study would be a minimum of 160. The survey will be available in the following languages: English, French, German, Italian, Spanish and Portuguese.

Why have I been invited?

You have been chosen to take part in this research because you are a General Practitioner (GP) who has at least one year of practice in primary care before and during the COVID-19 epidemic of 2020. You will have been identified by the study team and/or through GP networks and international organisations as someone

© Imperial College of Science, Technology and Medicine

who can speak of the impact of the pandemic on the implementation and use of digital-first models in primary care.

Do I have to take part?

It is up to you to decide whether or not to take part. If you do decide to take part, you will be given this information sheet to keep and be offered the option to provide consent before entering the survey. If you decide to take part you are still free to withdraw at any time and without giving a reason. If you choose to withdraw from the study, you can request that your data be excluded from future analysis.

What will happen to me if I take part?

If you decide to take part, you will be emailed a link to the survey and asked to read a shortened version of the Participant Information Sheet and provide consent before proceeding with the survey. The survey will take approximately 15 minutes to complete.

We will invite responses to the survey from June 2020 and seek to close the survey by the end of December 2020.

There is no follow up for this study, therefore you will only have to complete the survey once.

What do I have to do?

There are no requirements or restrictions to adhere to in order to participate in this study.

To take part in this study you must be over the age of 18 and able to communicate in written English, French, German, Italian, Spanish or Portuguese..

If you consent to participate in the survey you will be able to complete it in your own time while it is live.

What are the possible disadvantages and risks of taking part?

It is not anticipated that this study will cause any harm or risk to you.

What are the possible benefits of taking part?

While there are no expected benefits of taking part in this study, the resultant publications may highlight the positive contributions of DHTs to primary care as well as the areas for improvement that are needed to improve the long-term adoption of DHTs to support the delivery of primary care.

© Imperial College of Science, Technology and Medicine

What if something goes wrong?

If you are harmed by taking part in this research project, there are no special compensation arrangements. If you are harmed due to someone's negligence, then you may have grounds for a legal action. Regardless of this, if you wish to complain, or have any concerns about any aspect of the way you have been treated during the course of this study then you should immediately inform the Principal Investigator (Dr Ana Luisa Neves). If you are still not satisfied with the response, you may contact the Imperial Joint Research Compliance Office.

What will happen to the results of the research study?

The data will be stored and analysed in Imperial College London's Big Data Analytical Unit Secure Environment (BDAU SE), an ISO 27001 certified research environment. Only researchers involved in this project will have access to the data. Prior to being granted access, researchers will be required to pass GDPR and Information Security Awareness (ISA) training and will be required to agree, with possible disciplinary action for noncompliance, to BDAU SE standard operating procedures.No personal or sensitive information will be collected from you at any stage of the study.

Academic papers for publication may be produced based on the findings of the study.

For the publication of findings from the study, you will have the choice, before you enter the survey, to approve of or decline permission for any quotes you provide in the survey to be published alongside your institution or the country in which your practice is based. You would never be named in any reports/publications.

Who is organising and funding the research?

The research team is from the Institute of Global Health Innovation, Imperial College London.

There is no external funder of this study, therefore no one in the study will receive financial compensation for taking part.

Who has reviewed the study?

This study was given ethical approval by (Ruth Nicholson), Head of Department and Joint Research Compliance Office (JRCO).

Contact for Further Information

For any further information you can contact by email any of the researchers of this study:

© Imperial College of Science, Technology and Medicine

Principal Investigator: Dr Ana Luisa Neves

Research Fellow in Clinical Analytics and Patient Safety

Institute of Global Health Innovation,

Imperial College London

ana.luisa.neves14@imperial.ac.uk

Co-Investigator: Dr Saira Ghafur

Lead for Digital Health

Centre for Health Policy, IGHI

Imperial College London

saira.ghafur13@ic.ac.uk

© Imperial College of Science, Technology and Medicine

Imperial College London

TRANSPARENCY NOTICE

HOW WILL WE USE INFORMATION ABOUT YOU?

Research Study Title: An International Study on the Impact of COVID-19 on the Adoption of Digital-First Models in Primary Care

SETCREC number: 20IC5956

Imperial College London is the sponsor for this study and will act as the data controller for this study. This means that we are responsible for looking after your information and using it properly. Imperial College London will keep your data for:

- 10 years after the study has finished in relation to data subject consent forms.
- 10 years after the study has completed in relation to primary research data.

We will need to use information from you to conduct this research project.

This information may include your professional title and organisation. People will use this information to do the research or to make sure that the research is being done properly. We will keep all information about you safe and secure.

Once we have finished the study, we will keep some of the data so we can check the results. We will write our reports in a way that no-one can work out that you took part in the study.

LEGAL BASIS

As a publicly-funded organisation, we have to ensure that it is in the public interest when we use potentially identifiable information from people who have agreed to take part in research. This means that when you agree to take part in a research study, we will use your data in the ways needed to conduct and analyse the research study.

We will conduct scientific research in compliance with the law and the recommendations and guidance published by the UK Information Commissioner's Office (ICO).

INTERNATIONAL TRANSFERS

There may be a requirement to transfer information to countries outside the European Economic Area (for example, to a research partner). Where this information contains any potentially identifiable data, Imperial College London will ensure that it is transferred in accordance with data protection legislation. If the data is transferred to a country which is not subject to a European Commission (**EC**) adequacy decision in respect of its data protection standards, Imperial College London will enter into a data sharing agreement with the recipient organisation that incorporates EC approved standard contractual clauses that safeguard how the data you have provided is processed.

SHARING YOUR INFORMATION WITH OTHERS

For the purposes referred to in this privacy notice and relying on the bases for processing as set out above, we will share your anonymous data with certain third parties.

- Other College employees, agents, contractors and service providers (for example, suppliers of printing and mailing services, email communication services or web services, or suppliers who help

© Imperial College of Science, Technology and Medicine

us carry out any of the activities described above). Our third-party service providers are required to enter into data processing agreements with us. We only permit them to process your anonymised data for specified purposes and in accordance with our policies.

WHAT ARE YOUR CHOICES ABOUT HOW YOUR INFORMATION IS USED?

You can stop being part of the study at any time, without giving a reason, but we will keep information about you that we already have. We need to manage your records in specific ways for the research to be reliable. This means that we won't be able to let you see or change the data we hold about you.

If you agree to take part in this study, you will have the option to take part in future research using your data saved from this study in the Big Data Analytical Unit Secure Environment (BDAU SE), Imperial College London.

WHERE CAN YOU FIND OUT MORE ABOUT HOW YOUR INFORMATION IS USED

You can find out more about how we use your information by asking a member of the study team or sending an email to Dr Ana Luisa Neves (ana.luisa.neves14@imperial.ac.uk).

COMPLAINT

If you wish to raise a complaint on how we have handled your data, please contact Imperial College London's Data Protection Officer via email at dpo@imperial.ac.uk, via telephone on 020 7594 3502 and/or via post at Imperial College London, Data Protection Officer, Faculty Building Level 4, London SW7 2AZ.

If you are not satisfied with our response or believe we are processing your data in a way that is not lawful you can complain to the Information Commissioner's Office (ICO). The ICO does recommend that you seek to resolve matters with the data controller (us) first before involving the regulator.

© Imperial College of Science, Technology and Medicine
