# Supplementary material for: Evaluating the Impact of COVID-19 on the Adoption of Virtual Care in General Practice in 20 Countries (inSIGHT): Protocol and Rationale Study
Source: JMIR Res Protoc. 2021 Aug 26;10(8):e30099. doi: 10.2196/30099 (PMC8396553; doi:10.2196/30099)
Supplement: Multimedia Appendix 2 [file resprot_v10i8e30099_app2.pdf]

English (United Kingdom)

Block 1

Participant Information

You are being invited to take part in a survey to help us better understand the use of digital-first models to deliver care across various international healthcare settings. The aim of this survey is to explore the international impact of COVID-19 on digital-first models in primary care**.** *Digital-first models are defined as contacts with healthcare that happen firstly by an online means (i.e. telephone, video, website,...) as opposed to the traditional face-to-face care.*

In this research study we will use information provided by you. Everyone involved in this study will keep your data safe and secure. We will also follow all privacy rules. At the end of the study we will save some of the data in case we need to check it and/or for future research. We will make sure no one can work out who you are from the reports we write.

The Participant Information Sheet tells you more about this, to view a copy of it please click here.

The survey will take approximately 15 minutes to complete. You can save your responses and come back to them later if you need to.

If you have any questions, please do feel free to contact the Principal Investigator of the study, Dr Ana Luisa Neves (ana.luisa.neves14@imperial.ac.uk).

- ☐ I confirm that I have read and understand the Participant Information Sheet (version 1.2 dated 28.05.2020) for the above study and have had the opportunity to ask questions which have been answered fully.
- ☐ I understand that my participation is voluntary, and I am free to withdraw at any time, without giving any reason and without my legal rights being affected.
- ☐ I consent to take part in this survey

Default Question Block

PART I: Participant and practice information

In this section, we aim to learn more about you and your clinical experience as a primary care physician. We aim to learn more about your practice, as well as your current use of digital technologies.

1A. Please select your gender

- Female
- Male
- Other
- Prefer not to answer

1B. Please select your age from the categories below:

- Under 30
- 30-39
- 40-49
- 50-59
- 60-69
- 70+
- Prefer not to answer

1C. For how long have you been working as a General Practitioner/Family Doctor?

- < 5 years
- 5 - 10 years
- 10 - 15 years
- 15 - 20 years
- > 20 years

1D. On average, how many hours of clinical work do you have per week?

1E. Are you involved in teaching activities? (i.e. teaching trainees or medical students, or affiliated to a university)

- Yes
- No
- Prefer not to answer

1F In which country do you currently practice?

[▾]

1G. In which type of setting is your practice located?

- Urban
- Rural
- Mixed

1H. In your practice, do you have access to electronic health records?

- Yes
- No

1I. For how long have you been using an electronic health record in your practice?

- Only after COVID-19 outbreak
- Before COVID-19 outbreak, but for less than 2 years
- 2-5 years
- 5-10 years
- More than 10 years

1J. How often do you personally access electronic health records in your practice?

- Less than once a month
- At least once a month
- At least once a week
- More than once a week
- Everyday

1K. Please tell us if your electronic health record system has any of the following core functions?

	Yes	Partially	No
Accessing to new and past test results by providers in multiple settings	○	○	○
Entering and storing orders for prescriptions, tests, and other services	○	○	○
Reminders, prompts, alerts, and computerized decision-support systems (eg. screenings and other preventive practices, identify drug interactions, facilitate diagnoses and treatments)	○	○	○
Efficient, secure, and readily accessible communication among providers	○	○	○
Efficient, secure, and readily accessible communication between providers and patients	○	○	○
Tools that give patients access to their health records	○	○	○
Tools that provide interactive patient education	○	○	○
Tools to carry out home monitoring and self-testing of chronic conditions (eg. diabetes)	○	○	○
Computerised administrative tools, such as scheduling systems	○	○	○
Stores electronic data in a standardised way to quickly generate reports	○	○	○

1L. In order to help us understand the digital maturity of your electronic health record system, please tell us how much you agree with the following statements.

	Agree	Neutral	Disagree
Most healthcare providers in our practice use the digital system	○	○	○
Our organisation is ready to use the digital system correctly	○	○	○
We have the individual abilities needed to use the digital system correctly	○	○	○
Our digital system has the capability to communicate across services or with other systems	○	○	○

	Agree	Neutral	Disagree
We have best practice digital maturity evaluation methods in place	○	○	○
Our system has a positive impact in terms of outcomes for patients, structure, process or finance	○	○	○

1M. How do using electronic health records impact the following aspects of your practice?

	Positive impact	No change	Negative impact
Avoiding harm to patients from the care that is intended to help them.	○	○	○
Respecting and responding to individual patient preferences, needs, and values	○	○	○
Providing services based in scientific knowledge to those who could benefit and refraining services to those not likely to benefit	○	○	○
Avoiding waste of human and financial resources, equipment or supplies	○	○	○
Reducing waits and delays in delivery of care	○	○	○
Providing care that does not vary in quality because of the patient personal characteristics	○	○	○

Block 2

PART II: Use of digital-first models BEFORE and DURING the COVID-19 pandemic

On **11th March**, the World Health Organisation (WHO) declared the novel coronavirus (COVID-19) outbreak a global pandemic.

In this section, we will explore your experience using digital-first models (i.e. video and telephone consultations, remote monitoring, …) BEFORE and DURING the COVID-19 pandemic, as well as any training and guidance you might have received.

2A. Please select which technologies were available in your practice, before and during the COVID-19 pandemic (from 11th March).

Please select all which apply to these statements

	Available BEFORE the COVID-19 pandemic	Available DURING the COVID-19 pandemic	Not available
Remote access to patients' records from home	☐	☐	☐
Telephone consultations	☐	☐	☐
Chat consultations (ie. using a messaging system)	☐	☐	☐
Video consultations	☐	☐	☐
Online triage (e.g. telephone, video)	☐	☐	☐
Remote clinical monitoring technologies	☐	☐	☐
Patient-initiated digital services (ie. scheduling, health education activities, prescription renewal, test requests, sick leave)	☐	☐	☐
Secure messaging systems (ie. to communicate results to patients)	☐	☐	☐
Other *(please specify below)*:	☐	☐	☐

2B. On average, how many hours per week did you usually spend in the following clinical activities?

Please enter a number for BEFORE and DURING and put 0 for technologies that were not available at that time

	BEFORE the COVID-19 pandemic	DURING the COVID-19 pandemic
Remote access to patients' records from home		
Telephone consultations		
Chat consultations (ie. using a messaging system)		
Video consultations		
Online triage (e.g. telephone, video)		
Remote clinical monitoring technologies		

	BEFORE the COVID-19 pandemic	DURING the COVID-19 pandemic
Patient-initiated digital services (ie. scheduling, health education activities, prescription renewal, test requests, sick leave)	☐	☐
Secure messaging systems (ie. to communicate results to patients)	☐	☐

2C. Concerning training and guidance in digital-first technologies:
Please select all which apply to these statements; if not applicable please leave blank.

Digital-first models are defined as contacts with healthcare that happen firstly by an online means (i.e. telephone, video, website,...) as opposed to the traditional face-to-face care.

	Yes, BEFORE the COVID-19 pandemic	Yes, DURING the COVID-19 pandemic
Were you offered any training on digital-first technologies?	☐	☐
Did you undertake any training on digital-first technologies?	☐	☐
Are you aware of any local guidance advising the use of digital-first solutions?	☐	☐
Are you aware of any national guidance advising the use of digital-first solutions?	☐	☐
Were you aware of any guidance around cybersecurity and the use of digital-first technologies in healthcare?	☐	☐
Was there any specific guidance or training for family medicine / general practice trainees?	☐	☐

2D. I recommended the following digital health support solutions to my patients:
Please select all which apply to these statements - if not recommended, please leave blank.

	Yes, BEFORE the COVID-19 pandemic	Yes, DURING the COVID-19 pandemic
Symptom checker/self-assessment tool	☐	☐
Online information resources	☐	☐
Health trackers (e.g. FitBit, AppleHealth)	☐	☐

	Yes, BEFORE the COVID-19 pandemic	Yes, DURING the COVID-19 pandemic
Mindfulness apps/websites (e.g. The Mindfulness App, Headspace, Calm)	☐	☐
Online counselling services (e.g. BetterHealth, e-Therapy)	☐	☐
Online crisis resolution services (e.g. Shout)	☐	☐

Block 3

PART III: Experience and impact of using digital-first models DURING the COVID-19 outbreak

In this section, we will explore your experience and perceived impact of using digital-first models DURING the COVID-19 outbreak.

3A. How was your overall experience using digital-first technologies in the context of the COVID-19 pandemic?

Very Good	Good	Neither Good nor Bad	Bad	Very Bad
○	○	○	○	○

3B. In the context of the COVID-19 pandemic, how have each one of these technologies impacted patient care?

	Positive impact	No change	Negative impact
Remote access to patients' records from home	○	○	○
Telephone consultations	○	○	○
Chat consultations (ie. using a messaging system)	○	○	○
Video consultations	○	○	○
Online triage (e.g. telephone, video)	○	○	○
Remote clinical monitoring technologies	○	○	○

	Positive impact	No change	Negative impact
Patient-initiated digital services (ie. scheduling, health education activities, prescription renewal, test requests, sick leave)	○	○	○
Secure messaging systems (ie. to communicate results to patients)	○	○	○

3C. In the context of the COVID-19 pandemic, how have digital-first technologies overall impacted your clinical practice?

	Positive impact	No change	Negative impact
Reduction of the risk of transmission of COVID-19	○	○	○
Monitoring infected patients	○	○	○
Remote delivery of preventive care	○	○	○
Management of patients with chronic conditions	○	○	○
Remote delivery of acute care (ie. minor illnesses in primary care)	○	○	○
Communication and supporting the patient-doctor relationship	○	○	○
Communication between healthcare providers	○	○	○
Ability to gauge patients' emotional wellbeing	○	○	○
Patient satisfaction	○	○	○
Waiting lists and harmful delays in delivery of care	○	○	○
Gauging if patients require more input regarding care delivery	○	○	○
Identifying patients that need escalation in care	○	○	○
Providing equitable care for all	○	○	○
Avoiding waste of human and financial resources, equipment or supplies	○	○	○

3D. In your opinion, which were the biggest benefits of using digital technologies during the COVID-19 pandemic??

3E. In your opinion, which were the biggest challenges (i.e. problems an unintended harms) of using digital technologies during the COVID-19 pandemic? How did you overcome them?

Block 4

PART IV: Future of Digital-first Models in Primary Care

In this section, we will explore how the use of digital-first models during the COVID-19 pandemic can reshape the delivery of primary care, lessons learned and the way forward.

4A. After the COVID-19 outbreak, how would you LIKE the adoption of these tools to evolve in the future?

	Higher adoption than before COVID-19	Return to pre-COVID-19 adoption levels	Lower adoption than before COVID-19
Remote access to patients' records from home	◯	◯	◯
Telephone consultations	◯	◯	◯
Chat consultations (ie. using a messaging system)	◯	◯	◯
Video consultations	◯	◯	◯
Online triage (e.g. telephone, video)	◯	◯	◯

	Higher adoption than before COVID-19	Return to pre-COVID-19 adoption levels	Lower adoption than before COVID-19
Remote clinical monitoring technologies	○	○	○
Patient-initiated digital services (ie. scheduling, health education activities, prescription renewal, test requests, sick leave)	○	○	○
Secure messaging systems (ie. to communicate results to patients)	○	○	○
Any other technology *(please specify below)*:	○	○	○

4B. Can you list three main barriers that could lead to a return to pre-COVID-19 adoption levels or lower?

4C. After the outbreak is resolved, which are the TOP THREE aspects that are important to ensure a high-quality adoption of digital-first technologies in the future?

- ☐ Producing strong evidence of a positive impact of digital-first technologies
- ☐ Involving healthcare professionals and patients on the design and maintenance of digital-first solutions
- ☐ Improving usability for the end-users (i.e. healthcare professionals and patients)
- ☐ Providing digital training and support
- ☐ Producing clear guidelines and regulations
- ☐ Ensuring patient safety and privacy are protected
- ☐ Guaranteeing interoperability between different systems
- ☐ Improve technical infrastructure (i.e. hardware)
- ☐ Increasing investment on digital-first technologies
- ☐ Other *(please specify below)*

Powered by Qualtrics
